# Comparing Transcatheter and Surgical Mitral Valve Repair in Functional Mitral Regurgitation: Insights From a Real‐World Analysis and Future Research Directions

**DOI:** 10.1002/clc.70015

**Published:** 2024-10-04

**Authors:** Ibrahim Manzoor, Mustafa Mansoor

**Affiliations:** ^1^ Islamic International Medical College Rawalpindi Pakistan

To the Editor,

Functional mitral regurgitation (FMR) is characterized by normal mitral leaflet morphology but a dilated mitral annulus due to left ventricular dilatation or papillary muscle dysfunction [1]. The prevalence of FMR is increasing due to predicted demographic changes and it is estimated that by 2030, 4 million people will be diagnosed with FMR in the United States [2]. Patients with FMR are typically treated with guideline‐directed medical therapy (GDMT) but when symptoms occur despite GDMT, surgery or transcatheter intervention is indicated before left ventricular function further deteriorates. Recently, transcatheter edge‐to‐edge mitral valve repair (TEER) has emerged as a promising treatment modality in this regard. However, there is insufficient data comparing the effectiveness of TEER to the more commonly employed surgical mitral valve repair (SMVr).

To explore this gap, Wang et al. recently presented a real‐world analysis comparing the outcomes of TEER and SMVr in patients with FMR by using data from the National Inpatient Sample (NIS) database [3]. In 6233 and 2524 patients who underwent SMVr and TEER, respectively, the length of hospital stay was considerably shorter for TEER with a mean of 8.59 days for SMVr and 4.13 days for TEER. While the in‐hospital mortality rate was similar in both groups, the study showed that SMVr was associated with increased perioperative complications such as cardiogenic shock, cardiac arrest, respiratory failure, fluid and electrolyte disorders, acute kidney injury, cerebrovascular infarction, and bleeding after the procedure. Postprocedural blood transfusion and mechanical ventilation use were also higher in the SMVr group.

This comprehensive study provides valuable data and a nuanced understanding of the comparative outcomes between transcatheter and surgical approaches to mitral valve repair. The meticulous analysis and detailed presentation of results significantly contribute to the field of cardiovascular medicine, offering a robust foundation for future research and clinical practice. For certain patients with anatomical features that make TEER challenging or not feasible, and where surgical risk remains high and prohibitive, transcatheter mitral valve replacement (TMVR) has recently emerged as an attractive option [4]. While a number of transcatheter replacement devices are under development and clinical investigation, their current use is largely limited to compassionate cases or clinical trials. Consequently, TEER is the first‐line transcatheter treatment for both mitral and tricuspid regurgitation in many patients who are not candidates for surgery [5].

However, several limitations should be addressed in future research to enhance the robustness of the findings. The study's lack of laboratory or echocardiography data is a notable limitation, and subsequent studies should incorporate these assessments both pre‐ and postsurgery. Conducting randomized controlled trials with standardized data collection protocols across multiple centers will provide more robust evidence, aiding in the development of evidence‐based guidelines for managing mitral valve disease. Additionally, the absence of specific information on the transcatheter mitral valve repair (TMVr) devices used, as well as data on resource utilization and procedural trends, limits the study's applicability. Specification of the devices used and detailed information on resource utilization and trends by region and time will help provide better insight. The lack of subgroup data on TMVr, which is generally riskier and used for specific clinical and anatomical reasons, is another significant limitation. Future studies should include detailed subgroup analyses to better understand TMVr outcomes and tailor treatments to specific patient populations. Lastly, the NIS database's lack of longitudinal tracking prevents the assessment of long‐term outcomes. To address this issue, databases capable of longitudinal follow‐up should be used to provide a comprehensive understanding of the long‐term efficacy and safety of both TMVr and SMVr. Addressing these limitations will significantly enhance the quality and applicability of future research in this critical area of cardiovascular medicine.

## Author Contributions

Ibrahim Manzoor came up with the idea for the letter and wrote the manuscript. Mustafa Mansoor helped in finalizing the manuscript and provided supportive ideas for its completion.

## Conflicts of Interest

The authors declare no conflicts of interest.

## Data Availability

Data sharing is not applicable to this article as no new data were created or analyzed in this study.
